# Intra-Arterial Chemotherapy: New Hope for Adult Retinoblastoma Treatment—A Case Report and Brief Review

**DOI:** 10.1155/2021/6639900

**Published:** 2021-07-09

**Authors:** Hamid Riazi-Esfahani, Babak Masoomian, Fariba Ghassemi

**Affiliations:** ^1^Eye Research Center, Farabi Eye Hospital, Tehran University of Medical Sciences, Tehran, Iran; ^2^Ocular Oncology Department, Farabi Eye Hospital, Tehran University of Medical Sciences, Tehran, Iran

## Abstract

**Background:**

Adult retinoblastoma (RB) is a rare intraocular tumor, leading to delayed diagnosis and, eventually, enucleation of the involved eyes. Therefore, this diagnosis should be considered if attributable signs and symptoms occurred. Here, the authors report a case of an adult group D RB, treated successfully with intra-arterial chemotherapy (IAC) as primary treatment followed by cryotherapy. The authors briefly review the literature on the prognosis and management of adult RB. *Case Presentation*. A 22-year-old man has noticed floaters in his right eye for 2 months. Right eye examination revealed diffuse white spherical calcified vitreous seeds in all quadrants and a large white endophytic mass in the superonasal quadrant with engorged feeding vessels. Based on clinical data, the group D RB tumor was classified and the IAC was started with 2 medications, melphalan (5 mg) and topotecan (1 mg), for 3 cycles. Trans-scleral triple freeze-thaw cryotherapy was used as an adjunctive treatment at the latest hospital visit. Thirteen months after the last treatment, the RB tumor showed type 4 regression (flat chorioretinal scar) and no evidence of recurrence was identified.

**Conclusion:**

It seems that IAC, as the first line of treatment, either alone or in conjunction with adjuvant therapies may allow us to salvage the globe of adult patients involved with RB.

## 1. Introduction

Retinoblastoma (RB) is the most common primary malignant intraocular tumor of childhood originating from the sensory retina [1]. In more than 95% of cases, this tumor develops before 5 years of age, and its occurrence in individuals older than 18 years, which is considered adult-onset RB, is almost unusual [1]. Owing to its rarity, it is often not taken into account in the differential diagnosis of an intraocular mass in adult, leading to delayed diagnosis and, eventually, enucleation of the involved eyes [2–5]. Based on a literature review, to date only in three patients with adult-onset RB, the affected eye had been salvaged [2, 4, 6–8]. Adult RB is a diagnostic dilemma, and a high level of clinical suspicion is needed when faced with white intraocular mass in the adult's eye [4, 8].

Although intra-arterial chemotherapy (IAC) is increasingly used as a successful and safe treatment for childhood retinoblastoma, especially in unilateral cases, there are very few reports of this treatment modality used in adult retinoblastoma [6, 7]. Herein, we present a case of an adult with group D retinoblastoma, treated successfully with IAC as primary treatment followed by cryotherapy.

## 2. Case Report

A 22-year-old white man has noticed floaters in his right eye for 2 months.

**Figure 1:**
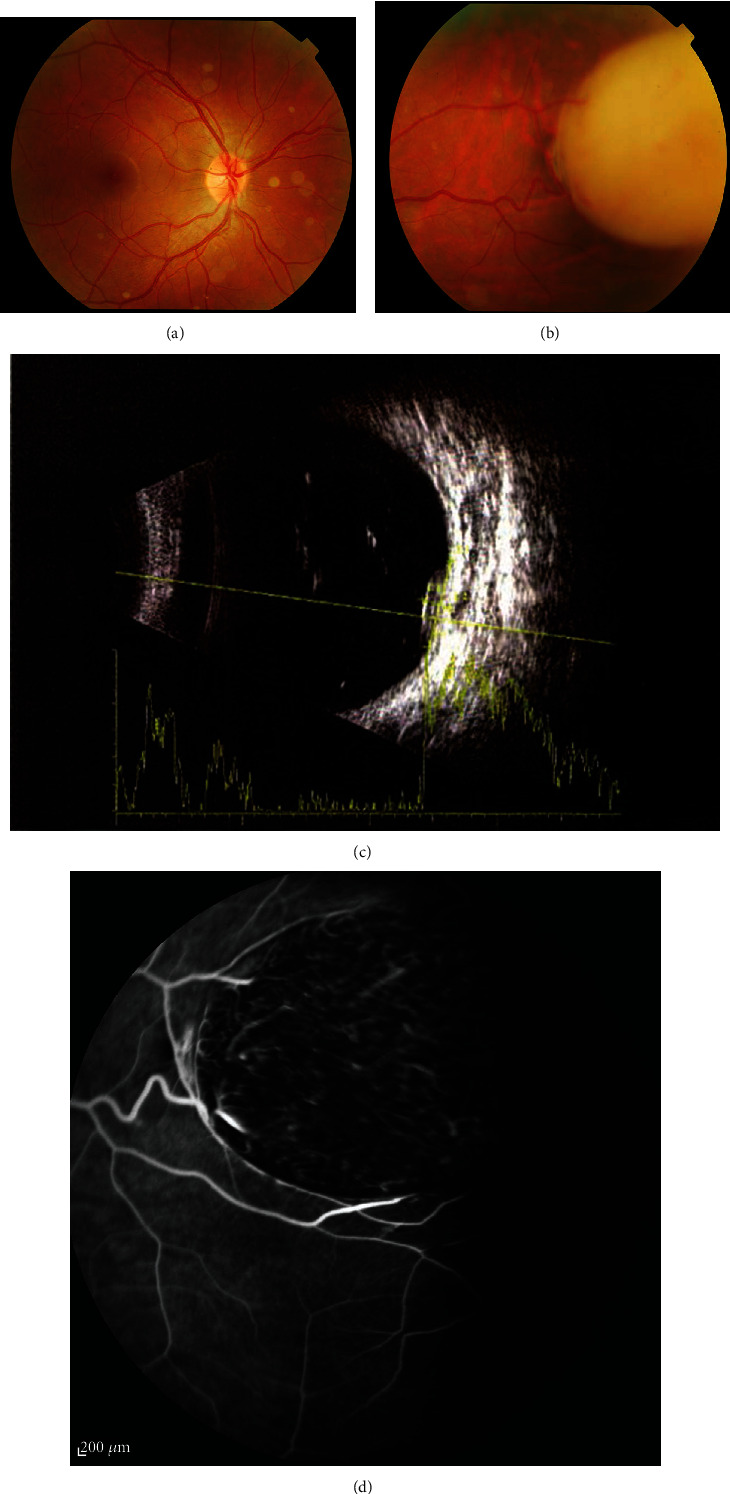
Right eye funduscopy revealed a white mass in the superonasal quadrant with engorged feeding vessels as well as diffuse white calcified vitreous seeds in all quadrants: (a) posterior pole and (b) the lesion site. In B-scan, the lesion had heterogeneous reflectivity by 8 × 7 mm in base and 3 mm in thickness. There were also some refractive vitreous debris compatible with calcified seeds (c). Fluorescein angiography (FA) showed engorged feeding vessels as well as intrinsic tiny vessels of the tumor (d).

**Figure 2:**
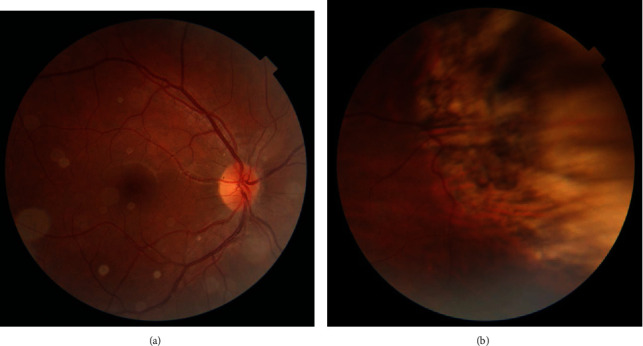
Thirteen months after the last treatment, the tumor showed type 4 regression (flat chorioretinal scar) and no evidence of recurrence. All the vitreous seeds were calcified: (a) posterior pole and (b) the lesion site.

He had no remarkable past medical history and no family history of RB. On presentation, the best-corrected visual acuity was 20/25 and 20/20 in the right and left eyes, respectively. Intraocular pressure was 16 mmHg in both eyes. Ophthalmic examination of the left eye was completely normal. Examination of the right eye revealed diffuse white spherical calcified vitreous seeds in all quadrants and a large white endophytic mass in the superonasal quadrant with engorged feeding vessels (Figures 1(a) and 2(b)). The lesion had heterogeneous reflectivity by 8 × 7 mm in base and 3 mm in thickness with some reflective vitreous debris in B-scan. There was no surrounding subretinal fluid (Figure 1(c)).

Fluorescein angiography (FA) showed engorged peripheral feeding vessels as well as the intrinsic tiny vessels of the tumor which had a slight leakage in the late phase of FA (Figure 1(d)).

Based on clinical data, the group D RB tumor was classified as International RB Classification (ICRB) and the IAC was recommended. He underwent three IAC cycles with 2 medications, melphalan (5 mg) and topotecan (1 mg), with an interval of 5 weeks between procedures.

IAC was done by using this protocol: under general anesthesia, intravenous heparin (50 IU/kg body weight) was instilled. Topical phenylephrine was applied locally along the distribution of the supratrochlear artery to minimize chemotherapy flow onto the forehead. The femoral artery of the ipsilateral side was accessed under aseptic precaution with an arterial sheath. This was carefully conducted under fluoroscopy up the aorta, then to the internal carotid artery, and then to the ostium of ophthalmic artery selectively. Following an angiography to ensure catheter placement at the ophthalmic artery ostium, each chemotherapeutic agent was diluted with saline in a 10 ml solution and injected in a pulsatile fashion throughout 10 minutes for a total infusion time of 20 minutes.

One month after the last IAC, the lesion showed an appropriate response with type 3 regression and 30% calcification, total calcified vitreous seeds, and 20/25 visual acuity in the treated eye. Trans-scleral triple freeze-thaw cryotherapy was used as an adjunctive treatment to the tumor at the latest hospital visit.

Thirteen months after the last treatment, the RB tumor showed type 4 regression (flat chorioretinal scar), and no evidence of recurrence was identified. Visual acuity was still 20/25 in the right eye. There was no sign of treatment complications to the surrounding normal retina as well as the optic nerve and the retina vessels (Figure 2).

## 3. Discussion

Since 1919, when the first case of bilateral RB was recorded by Maghy [9] in a 20-year-old young woman, isolated case reports and small case series of retinoblastoma have been reported in adults, all of which were unilateral [4, 5]. It is speculated that a persistent embryonic retinal cell with RB1 mutation can be a source of these tumors [2]. Despite significant advances in childhood RB treatment, our knowledge of adult RB is limited due to the rarity of the disease.

Over the last decade, the number of such cases has increased significantly, reflecting an improvement in suspicion and awareness among ophthalmologists [4]. A literature search using the PubMed/PubMed Central, Google Scholar, EMBASE, Scopus, and Cochrane databases has found only 50 reported cases to date. Adult records of retinoblastoma indicate more advanced cases (group D or E) that also require primary enucleation or even exenteration [2, 5]. The globe has been salvaged in only 4 adult RB patients, including our case, based on the literature so far [2, 6, 7] (Table 1).

Masoomian et al. demonstrated that in older patients compared to younger ones, tumors were more peripheral, with a greater mean distance from the optic nerve and foveola. Moreover, RB in older patients mostly presents with unusual symptoms and signs (e.g., floater or decreased vision). As a result, the unusual appearance triggers a late diagnosis and these cases are usually diagnosed clinically in advanced stages [10].

Misdiagnosis of the disease is common. In Kaliki et al.'s study, they found that about one-third of their adult RB patients had previously been misdiagnosed with other pathologies [2, 3].

Most published cases of adult RB have been managed primarily with enucleation [2, 4, 5]. In 11 cases who had undergone systemic chemotherapy or external beam radiotherapy (EBRT) or both, globe salvage could only be achieved in one case after EBRT [4]. To the best of the authors' knowledge, in 3 other cases (including our patient), the globe has been preserved successfully, in which all have undergone IAC, either primarily or secondarily [6, 7].

In the first report, IAC was used as a secondary treatment following failed EBRT [7]. Recently, McMahon et al. reported the first case of adult RB who had undergone IAC plus intravitreal chemotherapy as a primary treatment. The tumor showed complete regression, with complete resolution of intravitreal and subretinal seeds without any recurrence [6]. It seems that our present case is the second patient who has effectively undergone IAC as the first line of therapy.

IAC offers some benefits over systemic chemotherapy, with fewer probable systemic side effects [11]. In this process, higher doses of chemotherapy drugs (melphalan, topotecan, or carboplatin) are administered directly into the ophthalmic artery, resulting in a 30-fold rise in the concentration of chemotherapy agents at the tumor site [12].

Although IAC was associated with a slightly higher overall globe salvage rate than systemic chemotherapy, this advantage is significantly higher in eyes of Group D RB in comparison with other groups [11, 13]. As described before, adults with retinoblastoma typically have more advanced diseases [2, 5]. Therefore, IAC tends to be an acceptable alternative to initiate therapy.

Type 3 regression (defined as partially calcified tumor) has been shown to be a common regression pattern in adult-onset RB [14]. In our case, although the lesion showed an appropriate response to IAC with type 3 regression pattern, cryotherapy was used as an adjunctive treatment after IAC. As this type of regression is prone to recurrence, therefore, adjuvant therapies such as laser thermotherapy, cryotherapy, or radioactive plaques can be used to complete the treatment process along with IAC [11, 13].

The clinical and treatment characteristics of 4 adult RB cases that their eyes have been preserved are summarized in Table 1.

It is noteworthy that, increased awareness of intraocular lesions by ophthalmologists and the timely initiation of treatment have played an important role in saving the globe in adult RB patients in recent years.

In conclusion, newly diagnosed retinoblastoma may rarely occur in the adult population and appears to be advanced in diagnosis. In most cases, enucleation has been required. It seems that IAC, as the first line of treatment alone or in conjunction with adjuvant therapies, will be an effective treatment for adults with RB.

## Figures and Tables

**Table 1:** List of reported cases in which the eyes are salvaged with different treatment modalities.

Author	Year	Age/sex	ICRB group	Location	Tumor size (mm)	Growth pattern	Treatments	Final vision
Kaliki et al. [2]	2015	32/male	D	NA	NA	Endophytic	EBRT	NA
Magan et al. [7]	2016	32/male	D	Inferonasal	11 × 11 × 6.8	Endophytic	Primary treatment: EBRT	20/40
							Secondary treatment: IAC+brachytherapy+IVT	
McMahon et al. [6]	2019	23/male	D	Inferonasal	13 × 12 × 6.3	Endophytic	IAC+IVT	20/30
Riazi et al.	2021	22/male	D	Superonasal	8 × 7 × 3	Endophytic	IAC+cryotherapy	20/25

IAC: intra-arterial chemotherapy; IVT: intravitreal chemotherapy; ICRB: international classification of retinoblastoma; EBRT: external beam radiotherapy; NA: not available.

## Data Availability

The datasets used in the current study are available upon reasonable request.

## Abbreviations

RB: Adult retinoblastoma
IAC: Intra-arterial chemotherapy
FA: Fluorescein angiography
EBRT: External beam radiotherapy
ICRB: International RB Classification.
